# 

**Published:** 1809-04

**Authors:** 


### NEW MEDICAL PUBLICATIONS.

Observations on some of the most important Diseases of the Heart; on Aneurism of the Thoracic Aorta; Preternatural Pulsation in the Epigastric Region; and on the unusual Origin and Distribution of some of the large Arteries of the Human Body. Illustrated by Cases; by Allan Burns, Member of the Royal College of Surgeons, London, and Lecturer on Anatomy and Surgery, Glasgow.

Cases and Observations on Lithotomy, including Hints for the more ready and safe performance of the Operation. With an engraving. To which are added, Observations on the Chimney Sweepers' Cancer, and other Miscellaneous Remarks; by W. Simmons, Surgeon. 7s. 6d.

Anatomico-Chirurgical Views of the Nose, Mouth, Larynx, and Fauces; with appropriate Explanations and References; by John James, Surgeon. folio. 1l. 11s. 6d. plain, or 2l. 2s. coloured.

### To CORRESPONDENTS.

Mr. Birch's Letter referring to transactions in which the Journal has taken no part, and to a subject on which the Editors have for some time given notice to their Correspondents, that they wish to contract the space it once occupied, that Gentleman will not be surprised if we decline its insertion.

Mr. Blair's Letter being in answer to what he conceives personalities to himself, contained in our Journal, shall be inserted, if he chuses to confine himself to the inquiries concerning the person alluded to by the author, and the meaning of his words as they are printed in our text.

Dr. Roberton may depend on due attention being paid to his communications; but the number of favours we have received from our various Correspondents, prevents our alloting more than a certain space to each. Dr. Roberton must also correct the severity of his stile.

We are obliged to Mr. Hill for his ingenious communication, but fear it will be to long for such of our readers as are unconnected with Liverpool or its neighbouring cities. We will either shorten or return it, as may be agreeable to the author.

Our Correspondent Philo of Liverpool, will, we believe, find an ample account of the disease he refers to in the Manchester Controversy on the phlegmasia dolens. Should that not be the case, we will insert his quere.

Should we insert the question sent us by the learned Doctor of Winchester, we are not sure whose folly would be most exposed. If this Gentleman wishes to inform our readers, as well as to reform our Correspondents, we recommend him to employ his leisure in answering Senex's Letter contained in our last.

We are obliged to W. D. for his Paper, which shall be inserted in our next.

Dr. Pole's Eudiometrical, &c. Statements; Philanthropus's Paper on extensive Burns; and H's Letter, shall also appear in our next.

We are desired to correct the following Erratum in our last.—In Mr. Jenkinson's Letter, p. 327, instead of "the kind of rest, &c.;" read "the kinds of rest I am speaking of, accord so well with the indications (I wish I could add of cure), as set down by the best authors, and combine so easily with the remedies they have prescribed;" and after the words "of them" at the end of the same paragraph, to add "as auxiliaries."

